# Integration of Conductive Materials with Textile Structures, an Overview

**DOI:** 10.3390/s20236910

**Published:** 2020-12-03

**Authors:** Granch Berhe Tseghai, Benny Malengier, Kinde Anlay Fante, Abreha Bayrau Nigusse, Lieva Van Langenhove

**Affiliations:** 1 Department of Materials, Textiles and Chemical Engineering, Ghent University, 9000 Gent, Belgium; Benny.Malengier@UGent.be (B.M.); AbrehaBayrau.BayrauNigusse@ugent.be (A.B.N.); Lieva.VanLangenhove@UGent.be (L.V.L.); 2 Jimma Institute of Technology, Jimma University, P.O. Box 378 Jimma, Ethiopia; kinde.anlay@ju.edu.et; 3 Ethiopian Institute of Textile and Fashion Technology, Bahir Dar University, 6000 Bahir Dar, Ethiopia

**Keywords:** e-textile, integration technique, conductive material, smart textile

## Abstract

In the last three decades, the development of new kinds of textiles, so-called smart and interactive textiles, has continued unabated. Smart textile materials and their applications are set to drastically boom as the demand for these textiles has been increasing by the emergence of new fibers, new fabrics, and innovative processing technologies. Moreover, people are eagerly demanding washable, flexible, lightweight, and robust e-textiles. These features depend on the properties of the starting material, the post-treatment, and the integration techniques. In this work, a comprehensive review has been conducted on the integration techniques of conductive materials in and onto a textile structure. The review showed that an e-textile can be developed by applying a conductive component on the surface of a textile substrate via plating, printing, coating, and other surface techniques, or by producing a textile substrate from metals and inherently conductive polymers via the creation of fibers and construction of yarns and fabrics with these. In addition, conductive filament fibers or yarns can be also integrated into conventional textile substrates during the fabrication like braiding, weaving, and knitting or as a post-fabrication of the textile fabric via embroidering. Additionally, layer-by-layer 3D printing of the entire smart textile components is possible, and the concept of 4D could play a significant role in advancing the status of smart textiles to a new level.

## 1. Introduction

Clothing has been one of the three basic human needs since the beginning of our species. In the primitive age, textile was used for clothing purposes and progressively extended to household and domestic applications. Textile was also used for technical applications such as sailcloth, tent, protective garments, ropes, etc., which leveraged the textile properties to create a technical performance advantage.

**Figure 1:**
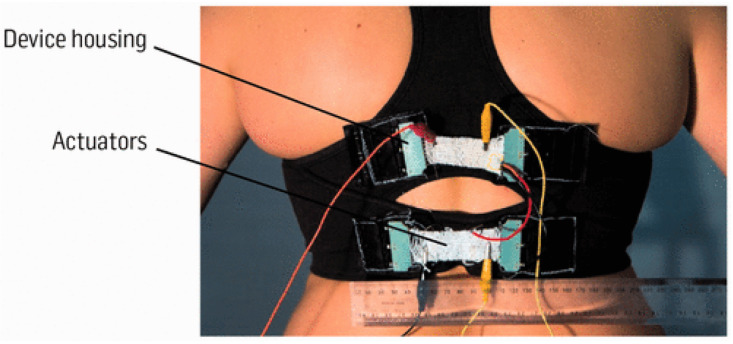
Bionic bra [3].

Smart textiles are materials and structures that sense and react to environmental conditions or stimuli, such as those from mechanical, thermal, chemical, electrical, magnetic, or other sources [1]. Textiles are materials that can react on themselves, unlike ordinary clothes. The expressions of “smart” and “intelligent” textiles or “wearable electronic” textiles, are commonly used interchangeably. The term “smart textile” may refer to either a “smart textile material” or a “smart textile system”. The definition is determined only by the context. Smart (intelligent) textile materials are functional textile materials actively interacting with their environment, i.e., responding or adapting to changes in the environment and smart (intelligent) textile system are textile system exhibiting an intended and exploitable response as a reaction either to changes in its surroundings/environment or to an external signal/input [2]. For instance, Steele et al. developed a bionic bra (Figure 1) using electro-material sensors and artificial muscle technology to detect the increase in breast motion and then respond with increased breast support to improve active living [3].

Smart textiles integrate a high level of intelligence and can be classified into three subgroups: passive, active, and very active or intelligent smart textiles [4]. They can be made by incorporating electronic materials, conductive polymers, encapsulated phase change materials, shape memory polymers and materials, and other electronic sensors and communication equipment. As Dadi 2010 studied, these materials interact according to their designed feature with the stimuli in their environment [5]. As an example of a very active smart textile, the first generation of wearable motherboards—which has sensors integrated inside garments that can detect injury and health information of the wearer and transmit such information remotely to a hospital—has already been developed [6].

### Building Blocks of Smart Textile Systems

**Figure 2:**
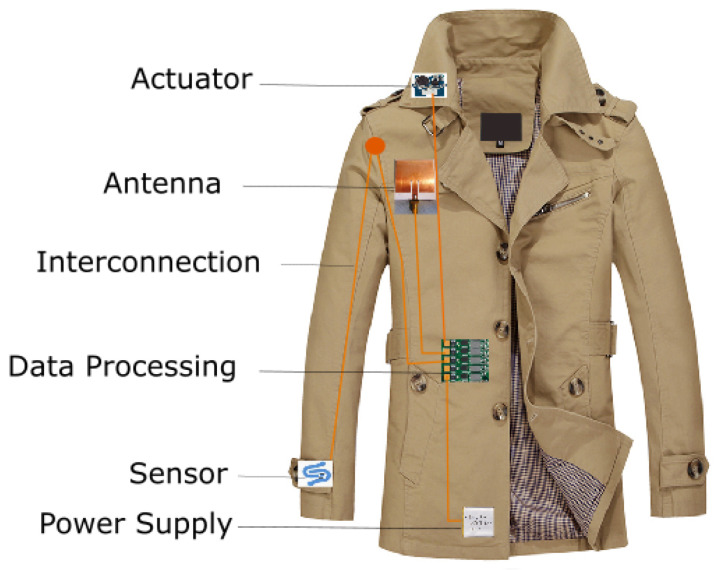
Building blocks of smart textile system.

Smart textiles with sensing and actuating capabilities for the desired use have been produced as a single purpose textile. However, the entire smart textile system could have specific function building blocks such as sensor, actuator, interconnection, controlling unit, communication device, and power supply. The schematic representation of a smart textile system is shown in Figure 2.

*Sensor:* A sensor is an electronic component that detects or measures a physical property and tracks and records, indicates, or otherwise responds to it. Typical textile-integrated sensor types include textile electrodes for strain [7,8,9,10,11], electrocardiography [12,13,14,15,16], electromyography [17,18,19], electroencephalography [20,21,22], humidity [23,24,25,26], temperature [27,28], pressure [28,29,30], light [31,32], and molecule detection [33,34].

*Actuator:* An actuator is a building component that can influence its environment. A typical use is to move or control other parts, but also light or sound generating parts are actuators. Common examples of textile actuators introduced are, organic light-emitting diodes [35,36,37], phase changing materials [5,38,39], temperature regulating textiles [40,41,42], and sound generating textile [43,44,45].

*Interconnection:* The interconnection is the part that links two or more functional components to one another. A lot of conductive textiles have been introduced for interconnection purposes [4,46,47].

*Control Unit:* The control unit is an electric board that directs the operation of the processor and is responsible for interpreting the signals from the sensor, ordering the actuator to react and commanding the communication device to transmit necessary messages. Specific examples of control units that can be integrated into textile system are Arduino [14,48,49], OpenBCI board [50], etc.

*Communication Device:* This is a unit integrated to transmit and receive electronic data and/or information from and to another system, respectively. A microstrip textile patch antenna [51,52,53,54] is a typical example.

*Power Supply:* The entire smart textile system must get the power to perform its task; the component included to provide power to the system is the power supply unit. For smart textiles, lithium polymer (LiPo) batteries are commonly used due to their size convenience. However, recently introduced textile-based energy harvesting devices [48,55,56,57] and storage capacitors [58,59,60,61] could replace these for some applications.

## 2. Search Method

A comprehensive electronic document search according to the PRISMA guidelines was conducted from February 2018 to September 2020 from the web of science in particular and Google databases, in general, using “building blocks of smart textiles” and “components of smart textiles” or “manufacturing of smart textiles” or “integration techniques of smart textiles” or “weaving + smart textiles” or “knitting + smart textiles” or “braiding + smart textiles” or “printing + smart textiles” or “embroidering + smart textiles” or “plating + smart textiles” or “coating + smart textiles” or “spinning + smart textiles” as keywords turn by turn. After duplicates were removed, 1166 articles remained. Articles were then screened by their title and abstract for relevance looking for a reference to integration techniques. After screening by title and abstract, we excluded 870 articles. Full text for the remaining 396 articles was accessed. Articles were included if they used any element of smart textile building block integration (including manufacturing options of smart textiles) and if the techniques used a textile-based and smart textile building block. However, articles were excluded if they were reviews, discussions, or commentary on integration and/or manufacturing of smart components, and if they did not use e-textile technology (most commonly these were studies using functional technology or wearable electronics). A total of 138 articles met the inclusion criteria and have been included in this review. It is worth noting that as e-textiles integration techniques are not entirely different from the conventional textile manufacturing and new ways of e-textile integration into a textile structure might still be under development, researchers with commercial links may have subsequent restrictions on publications of their findings, therefore, there may be some risk of bias in the studies found.

## 3. Conductive Materials for Textiles

Electrical conductive textiles are used in many applications of smart textile materials. However conventional textile materials are usually insulating materials, where they cannot be used directly for smart textile applications that require electrical conductivity. It is possible to obtain electrically conductive textile by integrating metallic wires, conductive polymers, or other conductive compounds into the textile structure at different stages, such as fiber construction, yarn spinning, or fabric creation stages. To impart conductivity, non-textile metallic filament wires made from silver, stainless steel, nickel, aluminum and copper can be inserted into the textile structure. Metals provide high conductivity which is very important for some smart textile applications but increases the weight of the material and affects their flexibility. Moreover, some metals are prone to corrosion. Apart from using metal wires, metal-based conductive textiles can also be produced by coating metal ink on the surface of textile materials, but these have limitations in wash stability. This leads to the search for alternative conductive compounds to produce reliable conductive textiles with better flexibility. Up to now, the conductive materials for textile materials can be categorized as conductive inks, carbon-based conductive polymers, intrinsically conductive polymers and conductive polymer composites.

### 3.1. Conductive Inks

The success of inkjet printing for printed electronics has attributed to the emergence of functional printable inks with different nanoscale sizes and structures. Based on their constituents, conductive inks can be categorized into three-dimensional nanostructured materials as nanoparticles, nanowires, nanotubes or they may exhibit plate-like shapes. The printable ink has a wide range of choices such as conductive, semi-conductive, and dielectric inks. The conductive inks can be prepared from conductive metal nano-particles and micro-particles. The semi-conductive inks can be prepared from metal-oxides, organic polymers and inorganic semiconductors. The dielectric inks are organic polymers in solvents, organic polymer thermosets or ceramic-filled organic polymers. Therefore, the functional conductive inks can be developed from metals, metal oxides, conductive polymers, organometallic inks, graphene, carbon nanotubes and a mixture of the different inks. Some examples of the conductive inks employed for the development of conductive textile are reactive silver [62], graphene ink [63], and carbon nanotube [64], etc. For instance, Liang et al. used a silver nanoparticle-based conductive ink that was configured with poly(styrene-block-ethylene-ran-butylene-blockstyrene) to develop a skin-inspired ultra-sensitive pressure sensor [65].

### 3.2. Carbon-Based Conductive Materials

**Figure 3:**
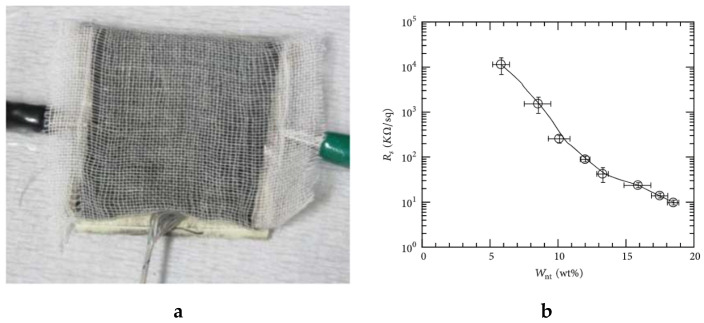
(**a**) Multi-walled carbon nanotube/cotton textile covered with one layer of cotton sheet; (**b**) the effect loading of multi-walled carbon nanotube in the cotton textile on sheet resistance [73].

As the need for conductive textiles gains importance, carbon-based materials such as graphene [66], carbon nanotube (CNT) [67], carbon black [68], graphene oxide [69], and reduced graphene oxides [70] have been investigated to develop electrically conductive textiles. These carbon materials are preferable for producing conductive textiles as most of them are relatively inexpensive, and they are corrosion-resistant and flexible [71]. In [72] graphene-based polyester conductive fabric was developed and used for bio-potential monitoring application. Rahman and Mieno have also developed an electro-conductive cotton textile by multiple dip-coating of the cotton fabric in a multi-walled carbon nanotubes solution. The surface resistance of the coated fabric decreased as the amount of carbon loading increased, which depends on the number of dippings [73]. The carbon-based conductive fabric is shown in Figure 3. Therefore, these materials can be used to produce a conductive textile with different ranges of conductance, up to more than 0.20 S/m depending on the load content. Other integration techniques like plating, transfer printing, inkjet printing, solution and electrospinning of carbon-based conductive materials could also provide a textile material with better conductivity and bulk property. For instance, Zhu et al. single-walled carbon nanotubes to fabricate machine-washable conductive textiles via dip-coating and spray coating [74]. The developed conductive textiles exhibit a high electrical conductivity of up to 7.4 × 10^2^ S/m.

### 3.3. Intrinsically Conductive Polymers

**Figure 4:**
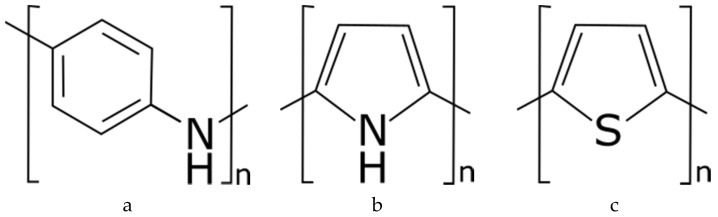
The most succeful conductive polymers: (**a**) polyanniline; (**b**) polypyrrole; (**c**) polythieophene.

At present, intrinsically conductive polymers are widely used in the development of electro-conductive textiles. Traditional organic polymers are electrical insulators or semiconductors, so the discovery of conductive polymers in 1970s [75], opened a new opportunity to produce electro-conductive textiles. Conductive polymers are polymers that contain a conjugated molecular structure that is having alternative single and double bonds between carbon atoms. They can combine the electrical property of metals or semiconductors with the benefit of conventional polymers such as price, structural diversity, flexibility and durability [76], which makes them an ideal choice for textile-based electrodes. Among the conductive polymers, polypyrrole (PPy), polyaniline (PANI) and polythiophene derivative poly(3,4-ethylene dioxythiophene):poly(styrene sulfonate) (PEDOT:PSS) are the most successful in the production of conductive textile [77]. The conductivity of the polymers can be enhanced by adding organic solvents called dopants, for instance, the conductivity of PEDOT:PSS can be enhanced from one to three orders of magnitude by adding polar organic solvents like ethylene glycol, dimethyl sulfoxide, glycerol [78,79,80,81]. Therefore, these conductive polymers can be used to develop all building blocks of the smart textile system as a wide range of electrical properties could be achieved by playing with the polymer add-on, and the extent of dopant. The chemical structure of some conductive polymers is shown in Figure 4.

### 3.4. Conductive Polymer Composites

Metal-based conductive textiles have the highest conductivity but are often not flexible enough. While, the existing conductive polymers show a promising conductivity, their mechanical properties need improvements. This has led to conductive polymeric composites with improved electrical conductivity and mechanical stability. Electrically conductive polymer composites are polymers consisting of single or hybrid conductive fillers such as carbonaceous, metallic, and conducting polymeric particles dispersed in a polymer matrix. They can be produced based on a single polymer or a multi-phase blend depending upon the electrical and mechanical properties required. Conductive polymer composites have been growing steadily and are being exploited for academic and industrial applications. [82,83,84,85]. As a result, a lot of conductive polymer composites have been introduced and used in developing conductive textiles. For instance, PEDOT:PSS-polydimethylsiloxane [84], PPy-silver nanocomposites [86], PANI-copper [87], graphene-PPy [88], PEDPT:PSS–CNT-Gr [89] have been reported as conductive polymer composites.

## 4. Integration Techniques of Conductive Materials on/into a Textile Structure

Smart materials are incorporated into the textile structure by different technologies; embroidering [90], knitting [91], weaving [92], spinning [93], braiding [94], coating [66], printing [84], plating [95] and chemicals that provide specific features such as controlled hydrophobic behavior [4]. The techniques of integrating a conductive material in/onto a textile structure can be categorized based on the form of the starting conductive material they use. The starting conductive materials can be conductive compounds, fibers, yarns, or sheets. The integrating techniques of these starting conductive materials are therefore different, we will present each of them in next sections.

### 4.1. Integration of Conductive Compounds

Conductive polymers and inks can be incorporated on textile materials by an in-situ polymerization of monomers on the textile substrate or by applying the conductive polymers and inks onto a textile substrate surface. In general, to produce a required e-textiles different approaches can be taken, such as adding the monomer, polymer or ink into a polymer solution during fiber spinning, during the coating/dyeing of textile substrates (fibers, yarns, fabrics) and/or in a printing stage on textile fabrics and garments.

In electrospinning, smart textiles can be also produced by adding sensor materials to a polymer spinning solution in nanotechnology level and microencapsulation and/or electrospinning technology. A process to encapsulate tiny particles or droplets into wall materials is quickly becoming a well-used technology for use in smart textiles [38]. Research is going into modifying fiber surfaces, such as grafting materials onto fibers to create multi-functional, responsive, and adaptive fibers, in order to tailor a hybrid nanolayer of polymer film that will afford several functions and properties through nanotechnology [96]. Printing is a common method to deposit a conductive layer on flexible fabrics and or garments fabric. Direct-write printing is defined as an additive manufacturing method in which the deposited patterns directly follow a pre-designed layout without utilizing masks or subsequent etching processes. Direct-write printing can deposit and pattern different thin film materials necessary for the fabrication of components and systems such as those found in electronic devices, sensors, and other systems. In the development of conductive textiles via printing, the conductive compound can be deposited or transferred to the textile substrate such as in screen and transfer printing, respectively. Inkjet printing and 3D printing can be potentially used to inject conductive materials over the surface of the textile fabrics layer by layer using a nozzle.

#### 4.1.1. Fiber Spinning

**Figure 5:**
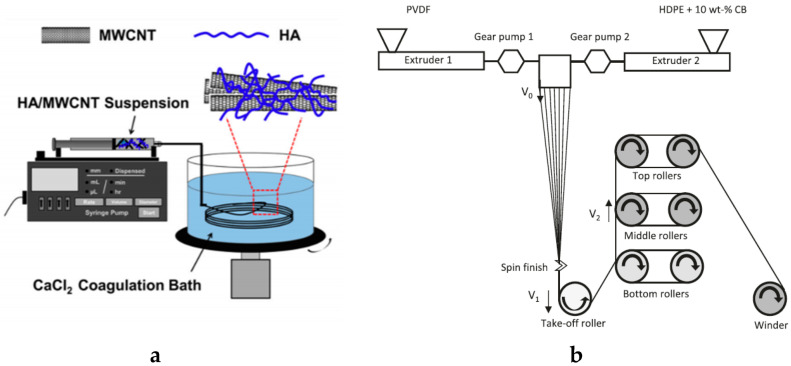
(**a**) A schematic of the experimental design of the wet-spinning method [93]; (**b**) schematic illustration of melt spinning [100].

Conductive components can be integrated into the textile structure at the fiber spinning stage by adding a conductive component into the polymer solution which are then extruded together to produce conductive fibers or filament. In [97], a conductive PANI fiber was reported using modified carbon black materials as conductive fillers via the wet-spinning process. The conductivity and tensile strength of the fiber were improved after annealing. Liu et al. developed electrically conductive composite fiber from a blend of PEDOT:PSS and PANI using a conventional wet-spinning process having a diameter of 30–60 µm. They reported that the electrical conductivity of the composite fiber increased as the content of PEDOT:PSS increased, where the highest conductivity was 5.0 S/cm when the PEDOT–PSS content was 1.83 wt % [98]. The PEDOT:PSS content does not show any relation to fiber mechanical performance, but it did cause an increase in surface roughness. Radzuan et al. also reported a PPy reinforced carbon fiber developed by the melt-spinning process [99]. The electrical conductivity of the fiber was 0.56 to 3.66 S/cm based on the die configurations. Zheng et al. used the wet-spinning method to fabricate hybrid microfibers composed of hyaluronic acid and multi-walled carbon nanotubes. The obtained hybrid microfibers presented excellent tensile properties with Young’s modulus of 9.04 ± 1.13 GPa and tensile strength of 130.25 ± 10.78 MPa, and excellent flexibility and stability [93]. Åkerfeldt et al. also used the melt spinning technique to produce a fully textile piezoelectric strain sensor, consisting of bi-component fiber yarns of β-crystalline poly(vinylidene fluoride) sheath and conductive high-density polyethylene/carbon black core as insertions in a woven textile, with conductive PEDOT:PSS coatings developed for textile applications [100]. The absence of the binder leads to one order of magnitude less in the surface resistivity, 12.3 Ω/square. However, the surface resistivity increases more upon abrasion when compared against fabric coated with binder added to the solution. Schematic illustration of the wet and melt spinning are shown in Figure 5.

#### 4.1.2. Dip-Coating

**Figure 6:**
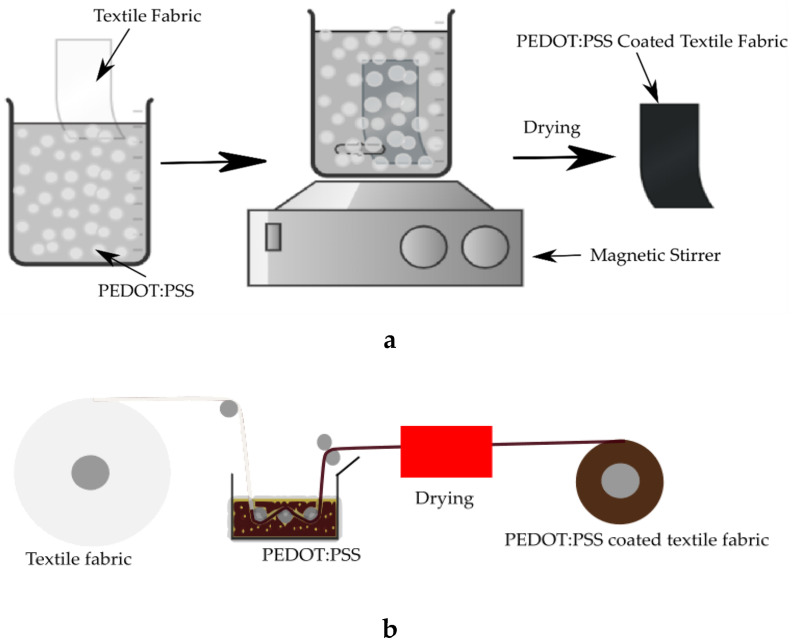
Methods of dip-coating: (**a**) discontinues process; (**b**) continuous process.

In this technique, textile materials such as fiber, yarn or fabric are immersed for a certain duration of time in a bath contain conductive dispersion. The process can be discontinuous or continuous as the schematics shown in Figure 6a,b, respectively. In the discontinuous process, the fabric is batched for some time in a solution containing conductive components and other auxiliaries. This method can be used for any form of textile. Whereas in the continuous process, a batch fabric passes through a padding mangle containing a conductive solution and drying unit as a roll. This method is suitable for fabric processing but can be used for a yarn too.

**Figure 7:**
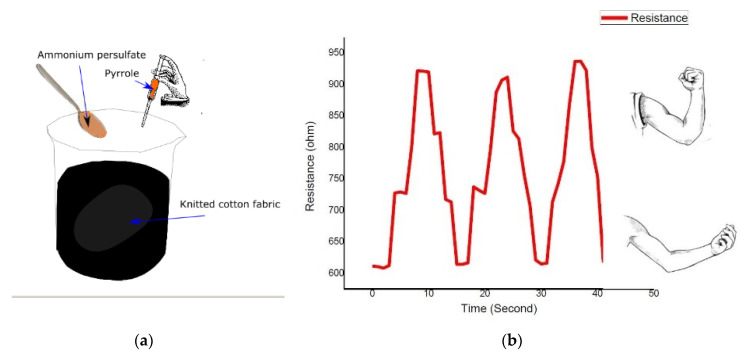
(**a**) Polypyrrole dip-coating of knitted cotton fabric; (**b**) strain response of the dip-coated fabric to bicep extension and flexion [103].

Many conductive textiles made by dip-coating have been reported. For instance, Liu et al. used the technique to fabricate a highly electrically conductive and excellent washing fast cotton fabric [95]. Liu et al. also reported a polypyrrole dip-coated electro-conductive cotton fabric developed by immersing the fabric in a solution containing polypyrrole at room temperature for 30 min [101]. The surface conductivity of the polypyrrole coated cotton fabric depended on the concentration of pyrrole in the solution, and better conductivity was obtained at 0.5 mol/L. Ankhili et al. dip-coated cotton, polyester and polyamide fabrics in a solution containing PEDOT:PSS dispersion. The cotton fabric gives better electrical conductivity because its good hydrophilic character caused a higher adsorption of PEDOT:PSS than the other fabrics [102]. Tseghai et al. have also used the dip-coating technique to develop a textile-based strain sensor by in-situ polymerization of PPy on cotton fabric. The sheet resistance of the dip-coated fabric was 60 Ω/sq [103]. The PPy dip-coated cotton fabric used for the construction of a strain sensor is shown in Figure 7.

**Figure 8:**
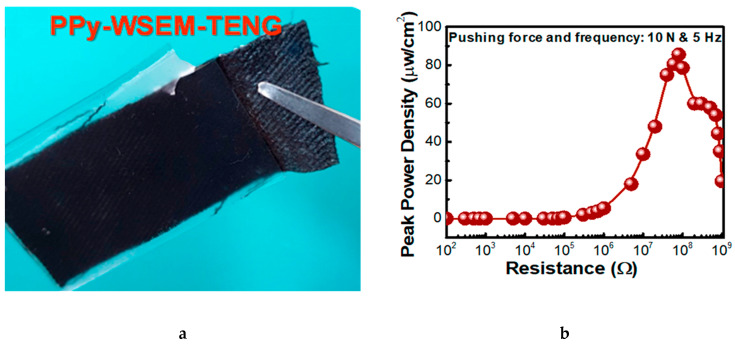
(**a**) Photographic image of a real polypyrrole-based wearable single-electrode-mode triboelectric nanogenerators (PPy- WSEM-TENG) device; (**b**) estimated peak power density values of the PPy-WSEM-TENG device under the applied pressing force and frequency of 10 N and 5 Hz, respectively [104].

Furthermore, Mule et al. used this technique to fabricate a conductive and robust PPy-coated cotton triboelectric nanogenerators (TENGs) device which was flexible and wearable [104]. The device efficiently converts mechanical energy into electricity while making a continuous touch-release with counter friction objects like human skin, i.e., tibo-friction layers. It shows strong characteristics even after long-term cyclic operations and conjointly produced an electrical yield under tender touching with the human hand. It can hence be utilized as a self-powered source to drive convenient electronic gadgets and light-emitting diodes. The photographic image of the PPy-based wearable single-electrode-mode TENG is shown in Figure 8.

#### 4.1.3. Plating

In this overview, plating is a process of adding a layer of *metal* components on the surface of textile materials. Before plating, the substrate must be cleaned to remove impurities, which offer assistance for effective attachment of the metal particles to the surface of the substrate [105]. There are different ways of plating, mostly categorized as electroplating or electroless plating.

**Figure 9:**
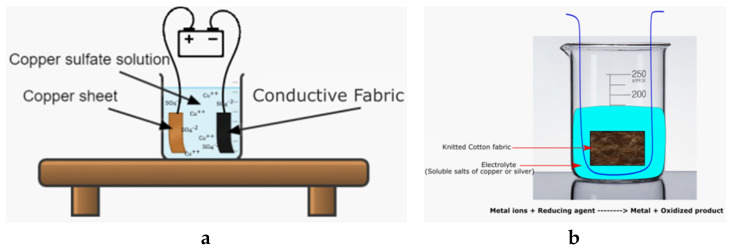
Plating methods: (**a**) electroplating, (**b**) electroless plating.

In electroplating, metals are plated on a surface of the conductive fabric by using electric current. A clean substrate can be immersed in a solution of metal plating particles and an electric current is applied to make the metals deposited to the surface. Hence, this way works only for conductive surfaces as otherwise the current cannot flow. The conventional textiles are not electrically conductive, therefore, it is not possible to use the electroplating, unless the material is prior conductive. However, this way can be used to improve the conductivity and stability of conductive textiles using metals particles. As an example of electroplated textiles, a carbon fiber electroplated with nickel for thermoelectric energy harvesting application was explored in [106]. A schematic representation of copper electroplating to a prior conductive cotton fabric is shown in Figure 9a.

Electroless plating is a chemical process to create metal coatings on various textile materials by an autocatalytic reaction, a chemical reduction of metal cations in a liquid bath. Electroless plating depends on chemical reaction to coat the metals on the surface of a material rather than using electric current. In this way, the plating is first performed by cleaning unnecessary components and impurities with chemical cleansers that are able to remove oils and other corrosive elements from the textile, then dip the substrate into an aqueous solution and add anti-oxidation chemicals. The schematic representation of electroless plating is shown in Figure 9b.

**Figure 10:**
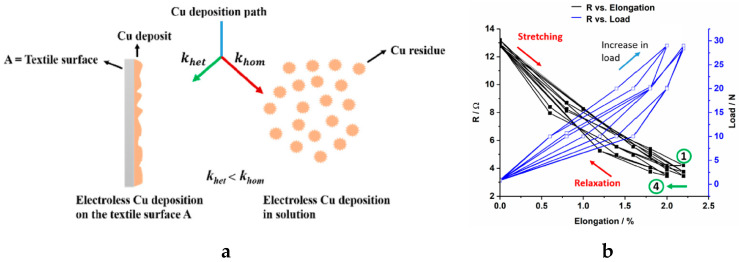
(**a**) A schematic drawing of the electroless copper deposition; (**b**) the electrical response to stretching [109].

Among the ways of plating, the electroless plating is more convenient for the traditional textile fabrics as it gives a high friction and corrosion resistance to the resulting conductive textiles, and can be used for non-conductive textile. Because of these, a lot of electroless plated conductive textiles have been developed and reported. For instance, Kumar and Thilagavathi developed a copper plated polyester fabric by using this technique. They reported that the plated polyester fabric provides good electrical conductivity with 300 kΩ/sq surface resistance [107]. Ma et al. used electroless silver plating to develop a conductive cotton/spandex blended fabric having robust electrical conductivity, 15.7 S/m [108]. The resultant fabric has high flexibility and stretchability due to the presence of spandex which could make it suitable as a strain sensor while obtaining an anti-bacterial property due to the presence of the silver. Therefore, this silver electroless plated textile could have a good potential prospect for wearable textiles. In addition, Root et al. reported copper electroless plated cellulose-based woven lyocell fabrics [109]. The resultant fabric was subjected to cyclic tensile tests; the resistance of the coated fabric (19 × 1.5 cm^2^) dropped from 13.2 to 3.7 Ω at 2.2% elongation. This work could attribute to a better understanding of conductive copper coating on textiles and their applicability as strain sensors. The schematic representation of the copper deposition and the electrical response to stretching of the fabric are shown in Figure 10.

#### 4.1.4. Screen Printing

As a feasible practical solution, screen printing is one of the most efficient and cost-effective methods of creating conductive patterns on different textile substrates. The screen-printing process consists of printing a viscous conductive paste through a patterned stencil followed by a curing process depending on the substrate and the property of the conductive compound used. It is among the highly recommended methods of printing since it can simplify the fabrication [53]. Therefore, screen printing is the widely used printing techniques [110] to realize textile electronics as one can easily deposit a pattern of conductive paste onto fabric to form a flexible strong and suitably thick functional layer after curing. The printing technique seems to be equal with dip-coating, but actually it is different. Printing is a structured application of the conductive components on a selected or localized area whereas, dip-coating is unstructured application of conductive materials to the textile material similar to the conventional dyeing of textiles.

Screen printing has powerful potential for manufacturing wearable electronics [111]. This technique can be used to apply conductive polymers and metallic and electrolyte inks onto textile substrates. For instance, screen-printed silver ink on cotton and polyester for ECG electrode [112], dispersion of carbon nanotubes on cotton and polyester fabrics [113], and PEDOT:PSS-based conductive polymer on cotton fabric [114] have been reported.

**Figure 11:**
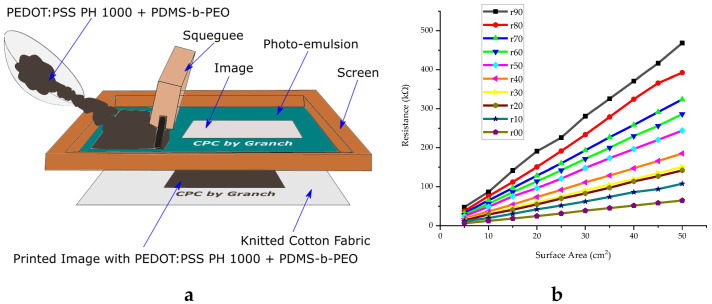
(**a**) Screen printing; (**b**) sheet resistance of the screen printed fabrics, width kept constant [84].

A lot of research effort is put into obtaining textile electronic components with smaller dimensions and with improved performance. It has been reported that electronic textiles like antennas have been screen printed on a polyester fabric and also transmission lines for RF and microwave systems have been screen printed on cotton [115]. Roshni et al. used the technique to develop an E-shaped microstrip patch antenna designed on polyester fabric for WiMAX applications [53]. Since the fabricated antenna was thin, flexible and water resistant, it can be easily integrated into any textile structure and garments which like this are able to sense and communicate data in a non-intrusive way. Liu et al. reported screen printed dye-sensitized solar cells (DSSCs) on woven polyester/cotton and Kapton fabrics for wearable energy harvesting applications. The screen-printed DSSCs on Kapton and polyester/cotton fabrics gave a photovoltaic efficiency of 7.03% and 2.78%, respectively [56]. Tseghai et al. exploited the screen printing of PEDOT:PSS- polydimethylsiloxane to develop a conductive cotton fabric [84]. A wide range of sheet resistance, i.e., 24.8 to 90.8 kΩ/sq was achieved by varying the proportion of the conductive polymer and elastomer. The schematic illustration of a typical flat screen printing is shown in Figure 11 together with sheet resistance results.

#### 4.1.5. Spray-Coating

**Figure 12:**
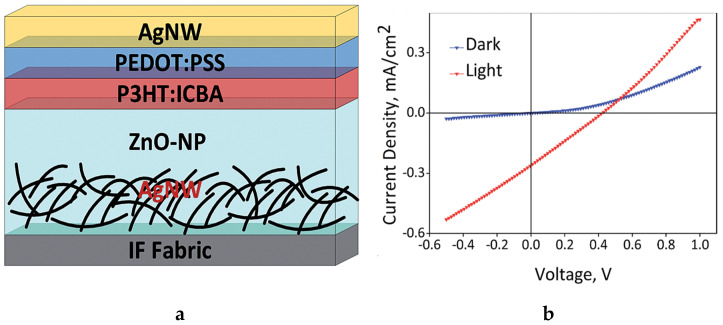
(**a**) Layer-by-layer spray-coated conductive polyester/cotton; (**b**) current density-voltage characteristics of the spray-coated conductive fabric [117].

In spray-coating, a spray of conductive particles or droplets are deposited to a textile substrate. A screen mesh or frame can be used to apply the conductive components at required locations. This technique has also extensively investigated in textile applications. For instance, Li et al. used the spray-coating to develop a textile based organic solar cells [116]. A power conversion efficiency of 0.4% was achieved. Arumugam et al. [117] also spray-coated silver single nanowire, zinc oxide nanoparticle, poly (3-hexylthiophene): indene-C60 bisadduct and PEDOT:PSS layer-by-layer on a woven polyester/cotton substrate for a solar cells as shown in Figure 12. A power conversion efficiency of 0.1% was achieved.

#### 4.1.6. Transfer Printing

In transfer printing, the required design is first printed on a non-textile substrate called print master and then transferred by a separate process to a textile fabric or garment upon the application of heat and pressure. This route would be chosen if direct printing on the fabric is not suitable. In most cases, such difficulties may arise from the rough surface of the textile fabric or the migration of the conductive component with solvent to undesired parts of the fabric due to the wicking effect. The particles can be transferred from the pre-printed master to the textile by sublimation, melt, film release and wet transfer.

The sublimation transfer is suitable for volatile compounds that can be preferentially adsorbed in a vapor phase by the textile material from a print master during heating. Though this method is commercially the most important of the transfer-printing methods, it is not well employed for the development of conductive textiles as volatile conductive compounds able to sublime during heating are not commercialized yet. The melt transfer can be used to print designs to a fabric with compounds that are able to melt on to the fabric in contact with the print master. This method is also not convenient for conductive polymers as they do not have a melting point. Metal conductive inks could be possible but commercial textiles could discompose before the melting point of the metal particles is reached. In the wet transfer, the design is transferred from print master to a moistened textile under a carefully controlled contact pressure. The conductive particles could then transfer by diffusion through the aqueous medium. However, the method is not used to any significant extent at the present time. The film release transfer printing is a little bit similar to melt transfer except the design is held in an ink layer which is transferred to the textile from a release paper using heat and pressure. Adhesion forces stronger than between the conductive film and the paper in the print master are developed between the conductive particles and the textile substrate. Therefore, this method can be potentially used to develop conductive textiles as the heating weakens the adhesion between the conductive film and the paper but not to melt the conductive components in the film.

**Figure 13:**
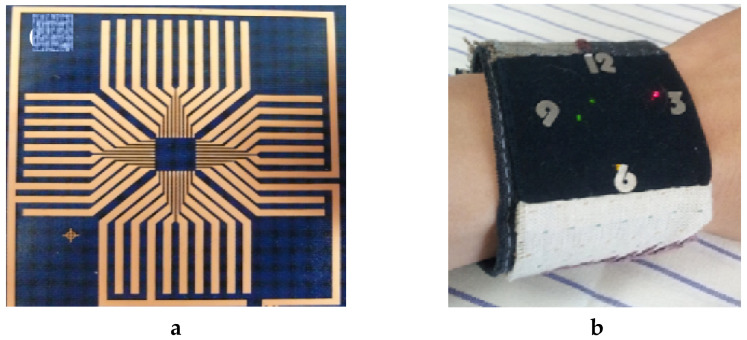
(**a**) Transfer-printed textile circuit; (**b**) textile wristwatch [119].

The film release transfer printing is already commonly used to develop conductive textiles. For instance, Maheshwari et al. used the film release transfer printing of silver nanowire conductive ink to textile fabric surface e-textile [118]. The resultant sheet resistance of the conductive fabric was small, i.e., 3 Ω/sq, and had a light weight and more mechanical flexibility than other conductive fabrics. Shin et al. also used transfer printing to develop a textile-based flexible circuit for a wristwatch that is shown in Figure 13 [119]. The transfer-printed textile circuit showed no change of resistance after folding while its equivalent screen printed circuit changed from 0.73 Ω/cm to 12.85 Ω/cm. Therefore, this is a promising integrating technique for any conductive particle in the form of a film to produce flexible and lightweight conductive textiles for different wearable applications.

#### 4.1.7. Inkjet Printing

Inkjet printing is a widely used direct-write deposition tool that has rapidly migrated to electronics fabrication in recent years. It is a key printing technique that has not been widely applied to wearable textiles fabrication. In inkjet printing, images and structures are built up in a droplet-by-droplet fashion. The user may make a change to the jetting parameters or to the ink. The technique of inkjet printing structures can be an advantageous manufacturing technique as the functional component can be created within minutes of finalizing the design; the finish is aesthetic and has excellent resolution; it requires minimal material consumption and as no mask is required there is the flexibility to change the design regularly. The process is an additive process that does not require environmentally harmful etching chemicals while minimizing the amount of waste produced and has a high degree of reproducibility as the droplet production allows a user to treat the droplets as building blocks. It is possible to print the ink directly on to the fabric, but Chauraya et al., 2013 argued that the pattern would dissipate into the textile and cannot produce a continuous conducting track without many layers being printed due to the high solvent content (~85%) of the inks required to ensure inkjet printability [115]. Inkjet printing requires the use of a special liquid, usually referred to as ink, which contains the smallest possible electrically conductive particles (their dimensions are usually counted in tens of nanometers at the most). For the stability of such a suspension in time, each of the conductive particles (mostly silver or gold) is covered with a protective organic layer. Carbon nanotube and graphene inks are also used but typically have lower conductivities than metallic inks [120].

**Figure 14:**
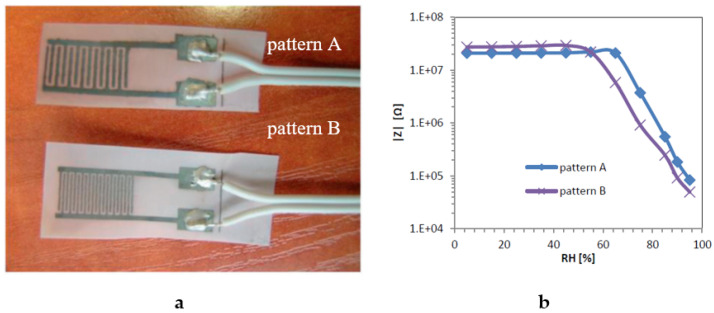
(**a**) A textile-based humidity sensor; (**b**) impedance modulus dependence on humidity for pattern at 1 kHz measurement frequency [24].

Al-naiemy et al. used the inkjet printing technique to develop a microstrip antenna based on nano-silver inkjet material [121]. The developed antenna operated more efficiently than its identical antenna made by screen printing at 2.44 GHz. This indicates that the integration of microstrip antennas, electronic circuits, and sensors to the panels of photovoltaic cells using inkjet printing is considered as a successful and promising design approach for the future. Inkjet printing of sol-gel derived tungsten inks on glass and transparent conductive tungsten oxide and functionality of these transparent WO_3_ layers were successfully demonstrated in an electrochromic device [122]. He et al. reported fully printed humidity sensors from graphene oxide and few-layered black phosphorus flakes dispersion printed silver nanoparticle electrodes via inkjet printing [123]. The sensor can give an electrical response from 11% to 97% relative humidity. In addition, the capacitance sensitivity was also high in both the graphene oxide (4.45 × 104 times) and the black phosphorus (5.08 × 103 times) sensor at 10 Hz operation frequency. Weremczuk et al. also used this technique to produce a textile-based humidity sensor that has satisfying metrological parameters [24]. This work demonstrated a prospective opportunity of integration with smart wearable electronics used for making medical applications. The photographic image of the humidity sensor and some results are shown in Figure 14.

### 4.2. Integration of Conductive Yarn and Conductive Filament Fiber

Conductive filament fibers, yarns, and metallic wires can be integrated into/onto a textile structure by weaving, knitting, embroidery, and braiding techniques. Though a conductive and functional e-textile can be developed via these techniques, the electrical and mechanical properties of the textile substrate could significantly vary from the initial conductive material. This is because of the way the conductive materials placed, the structure of the textile substrate, the density of the conductive fabrics on the substrate, and other factors that could potentially determine the end-product properties.

#### 4.2.1. Weaving

Weaving produces textiles that need work before they are usable in an end-product. The benefit is that there are more possibilities for integrating the active elements during the fabrication. As weaving typically utilizes a two-yarn system, i.e., it has a separate warp and weft, this naturally supports the use of different yarns. These can be varied, and even though looms require considerably more time and effort to set up, they seem to provide a reliable base for building electronic systems. As weaving is suitable for embedding electronic components into the textile during the weaving process, it also allows the encapsulation of the components between different layers. The woven textile forms a combination of thousands of threads in the warp and the weft. The warp has considerable tension, and warp threads move up and down during the weaving process, according to the harnesses they are connected to. The programmed pattern, which dictates how the threads connect within the weave, is realized with the weft. These yarns move orthogonally to the warp, and have typically low tension, with only the forces from the warp threads pressing against the weft.

For the case of developing e-fabric, conductive yarn or filament can be integrated as warp and weft. It is also possible to insert conductive threads along with non-conductive warp and/or weft yarns. Therefore, the pattern designs possible to produce a convention textile fabric could be used to produce an entirely conductive fabric or a fabric with incorporated conductive threads or wires.

**Figure 15:**
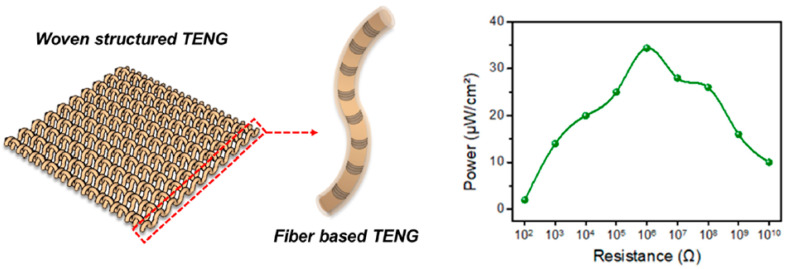
Schematic illustration of a fiber-based woven-structured triboelectric nano-generator (TENG) and its dependence of the output power on external load resistances [125].

Mikkonen and Pouta developed a wire component suitable for direct integration into the textile during weaving like a normal yarn and successfully demonstrated the weaving [92]. Gidik et al. used weaving technology to develop a textile heat fluxmeter [124]. The textile fluxmeter was used as a base to produce a textile radiative heat fluxmeter, i.e., to transform a textile heat fluxmeter to a textile radiative heat fluxmeter. Park et al. also used the weaving as a simple fabrication procedure to develop a flexible single-strand fiber-based woven-structured triboelectric nano-generator for self-powered electronics [125]. This device converts mechanical energy from living/working environments into electrical energy. The schematic illustration of the fiber-based woven-structured triboelectric nano-generator and its dependence of the output power on external load resistances is shown in Figure 15.

#### 4.2.2. Knitting

**Figure 16:**
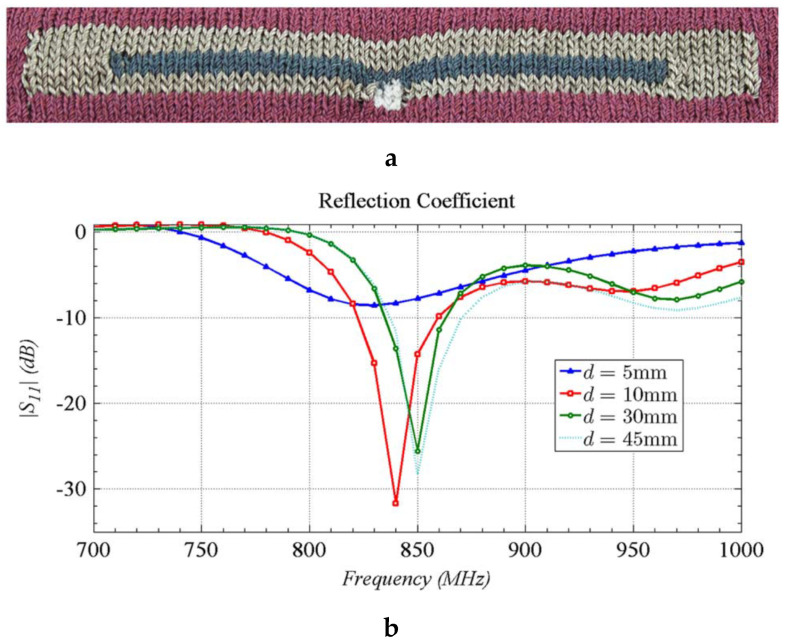
(**a**) The knitted e-fabric; (**b**) return loss of the radio-frequency identification (RFID) tag antenna for different distances [126].

Knitting is a continuous and efficient fabric manufacturing process. Apart from creating the textile as a substrate, knitting allows the inclusion of active elements and conductive yarns during the fabrication process, making them integral to the textile structure. As knitting costs relatively less to fabricate than weaving for small samples, it is a good candidate for the rapid prototyping of smart clothing and wearable textiles. In addition, the existing industrial knitting machines are also already able to create entire and complete knitted ready-to-wear structures. Recent advancements in conductive yarns and fabrication technologies offer exciting opportunities to design and knit seamless garments equipped with sensors. For instance, Patron et al. used this technique to produce a wearable antenna for wearable applications [126]. This knit antenna works as a strain sensor taking advantage of the intensity variations of the backscattered power from an inductively-coupled radio-frequency identification (RFID) tag under physical stretching. The actual image of the knit e-fabric and its return loss as an antenna is shown in Figure 16.

**Figure 17:**
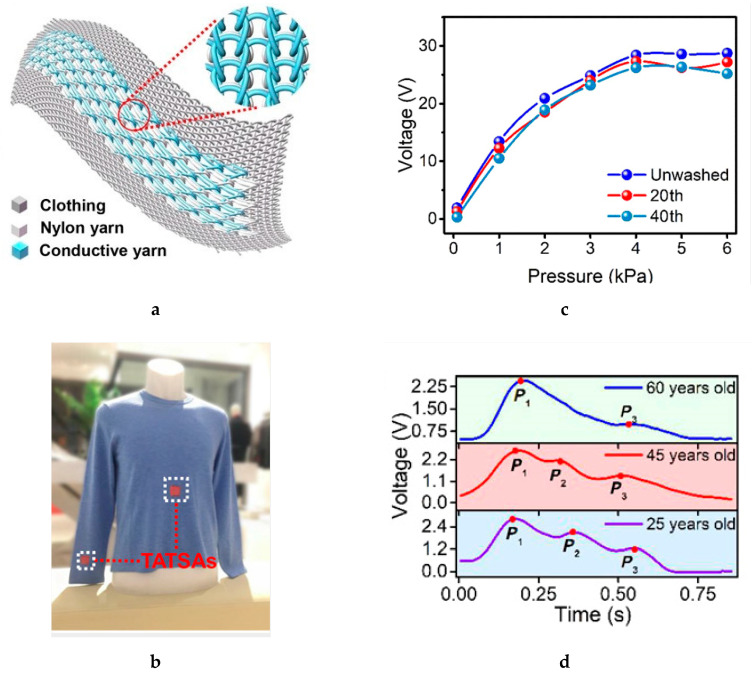
(**a**) Schematic illustration of the combination of triboelectric all-textile sensor array (TATSA): (**b**) photograph of two TATSAs completely and seamlessly stitched into a sweater; (**c**) output characteristics of the TATSA after washing; (**d**) pulse waveforms of TATSAs for different ages [128].

A computerized flatbed knitting method was also used to fabricate elliptical waveguide [91]. It is a conductive textile sleeve filled with knitted polyester inside. A silver-coated polyamide conducting yarn was used. The same technique was also used to manufactured a microwave high impedance surface from a combination of both conducting and insulating yarns [127]. The entire structure of the high impedance surface—the conducting ground plane, spacer layer, conducting pattern top surface and the vias—is knitted. Such a continuous development of an e-textile obviously has low cost and is highly efficient in terms of manufacturing time. Fan et al. also used the knitting technique to produce a machine washable textile-based triboelectric sensor array [128]. The sensor array exhibits a fast response time and wide working frequency bandwidth up to 20 Hz and stays functional for multiple machine washings. This textile-based sensor array was incorporated into a sweater as shown in Figure 17 to monitor the arterial pulse waves and respiratory signals simultaneously. The knitted triboelectric all-textile sensor array was also used to measure the cardiovascular pulse of different age groups.

#### 4.2.3. Embroidery

Embroidery is applying conductive yarns or filament fibers on a textile fabric or other materials using a needle. It gives the flexibility to design and embroider traces of required shapes or contours on a plane. Compared to other textile production technologies, such as knitting or weaving, embroidery is a convenient alternative for complex and labor-intensive design and production processes. This technique enables one to integrate additional conductive threads into a finished fabric or readymade garment. Embroidery has been exploited to develop e-textiles. For instance, Moradi et al. embroidered an e-textile metamaterial transmission line for a signal propagation control for wearable applications [129]. It was a fully-embroidered conductive thread transmission line loaded with conductive yarn split-ring resonators on a felt fabric substrate.

**Figure 18:**
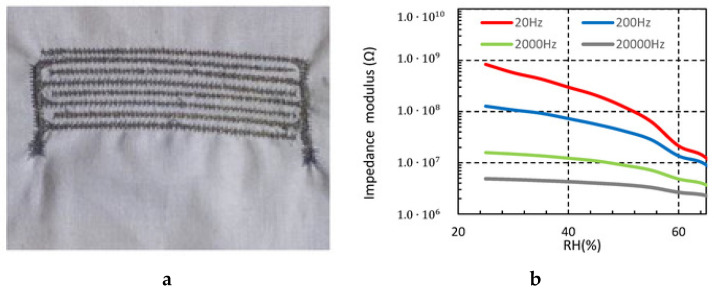
(**a**) Embroidered capacitive sensor; (**b**) measured sensor impedance from 25% to 65% relative humidity (RH) at different frequencies (T = 20 °C) [130] CC BY 4.0.

Martinez-Estrada et al. also used the technique to embroider an interdigitated textile sensor over a cotton substrate with silver-plated nylon yarns [130]. The result showed the usefulness of the proposed sensors at the kHz range to develop a wearable application over textiles for moisture detection as shown in Figure 18. Besides, Alharbi et al. introduced and validated a novel class of origami dipole antennas fabricated via adaptive embroidery of conductive e-threads [131]. A shift in resonant frequency from 760 to 1015 MHz was observed, while 84% of the original 10 dB bandwidth was retained, which shows an excellent agreement against a copper-based equivalent dipole.

**Figure 19:**
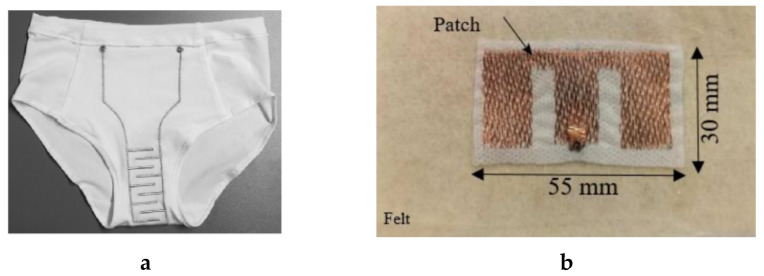
(**a**) Embroidered moisture sensor [46]; CC BY-NC-ND (**b**) e-shape antenna fabricated based on embroidering technique [132] CC BY 4.0.

Embroidering has been also used to develop a textile-based sensor and antenna as shown in Figure 19a,b, respectively. The technique seems very promising to produce an entire set smart textile as the sensors, actuators, capacitors, energy harvesting devices and interconnections can be embroidered step by step or one at a time.

#### 4.2.4. Braiding

**Figure 20:**
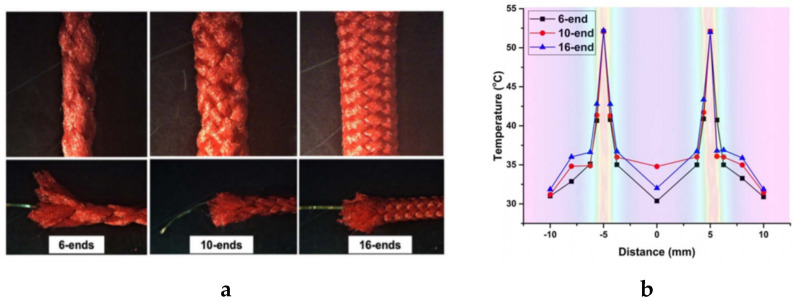
(**a**) Braided conductive yarns (BCYs) with single copper core covered with 6-, 10- and 16-end polyester sheath and 76°, 96°, and 120° braiding angles, respectively; (**b**) temperature variation around BCYs immediate environment [94] CC BY 4.0.

Braided conductive fabrics can be made by interlacing conductive yarns or strips of fabric. An entirely conductive braided fabric or partly conductive can be made. This technique produces a wide range of structures. For example, Pragya et al. used the braiding technique to produce a conductive yarn by introducing conductive copper filament as the core and polyester multifilament yarn as the sheath. The resultant braided yarn was used to fabricate an e-heating fabric via interweaving. The electro-mechanical tests on the braided conductive yarn and e-heating fabric revealed superior tensile performance and heat trapping with increasing the number of ends [94]. The braided conductive yarn and temperature variation around its immediate environment is shown in Figure 20. The braiding process is quite adaptable, however, there are certain inherent limitations related to the process itself, the input materials, geometry of the part, and the specific needs and standards for material characteristics and uniformity [133].

### 4.3. Integration of Conductive Sheets: Laminating

**Figure 21:**
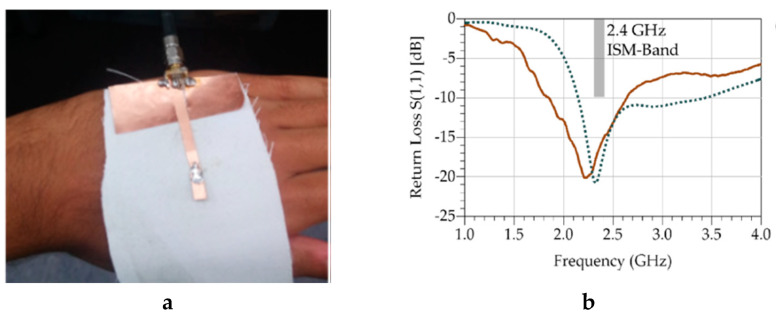
(**a**) Fabricated antenna on-hand; (**b**) simulated (dashed) and measured (solid) return loss showing broadband operation around 2.4 GHz and [137].

In this technique, a conductive sheet or stripe can be placed on textile fabrics by stacking and laminating via welding, an adhesive, or through the use of heat or pressure. Therefore, this technique can be used to produce e-fabric quickly. For instance, Vanveerdeghem et al. [134], Sorti and Company [135]. Choi et al. [136], etc., have reported e-textiles by conductive sheet lamination. As a specific example, Wagih et al. also used the technique to develop a textile-based patch antenna based on coplanar waveguide [137]. The efficiency of the coplanar waveguide monopole was independent of the thickness of the substrate and type of fabric. The fabricated antenna and the performance of the coplanar waveguide textile monopole are shown in Figure 21.

## 5. Outlook and Future Prospects

As aforementioned, a lot of techniques can be used to develop an e-textile. However, the standard textile production techniques may not be suitable to develop an e-textile of specific form. Weaving, knitting, embroidery, and braiding methods are nevertheless suitable to produce an e-fabric, or maybe a braided yarn in the case of braiding, by integrating a conductive yarn or filament fiber during the fabrication or post-fabrication. Coating and electroless plating on the other hand are convenient to produce all forms of e-textile by applying conductive polymers, inks, and their composites. However, the electroplating can only be used for prior conductive textiles. The melt and solution spinning method are suitable to produce e-fibers by adding conductive compounds or particles into the spinneret during the production of manmade fibers. Electrospinning can be used to fabricate e-fibers or e-webs on demand. The printing methods are convenient to produce an e-fabric. Lamination is suitable to place conductive sheets on a textile fabric. Therefore, all the methods can be exploited to develop required e-textile. However, the form of the textile, the form of the conductive material, the form of the required final product determines which technique to use.

Apart from that, all the methods have their own pros and cons [138]. The electrospinning allows the production of very thin fibers and webs with large surface area, however, it is problematic to obtain 3D structures or to control the pore structures. Wet spinning enables producing a wide variety of conductive fiber cross-sectional shapes and sizes, but, the method needs post-processing to remove impurities and solvents. Melt spinning has high production speed and is a simple process, but works only with thermoplastic polymers. Coating and plating methods are possible on any form of textile but they are slow and end with more waste. Weaving can obtain higher production rates but is more unreliable since it may sometimes cause wrinkling of the textile material. Machine embroidering is another successful technique to fabricate electronic circuits on textile substrates and garments, but it offers high tension in the yarn and causes yarn fraying which may adversely affect the quality of the circuit.

Functional electronic patterns can be easily laminated on to the textile substrate using suitable polymer adhesives but the high dielectric loss factor caused by the adhesive may also deteriorate the dielectric properties of the textile substrate, besides, delamination of the conductive film occurs during bending which is not recommended for flexible electronics [53]. Knitting is a fast fabrication method and can enable the fabrication of seamless stretchable e-fabrics but dimensional stability is poor. Braiding can be used to produce e-yarns and/or fabric for technical textile applications but results in a much heavier weight. Automatic screen printing enables to develop lightweight, flexible and foldable e-fabrics but much higher initial setup cost. Transfer printing enables textile printing to be carried out using simple and relatively inexpensive equipment with modest space requirements. Moreover, it allows us to produce complex designs more easily and accurately on paper than on textiles. Inkjet printing enables in-situ design and rapid printing of good quality complex designs but it needs special formulation of inkjet inks which are quite expensive. Ink bleeding and blockage of the printer nozzle is also a problem. In general, conventional textile manufacturing techniques are not sufficient enough for the production of e-textiles. Therefore, there is a high demand for more modified and improved integration techniques for electronic components on/into a textile structure. This needs a comprehensive integration among textile, electrical, mechanical, and chemical experts.

**Figure 22:**
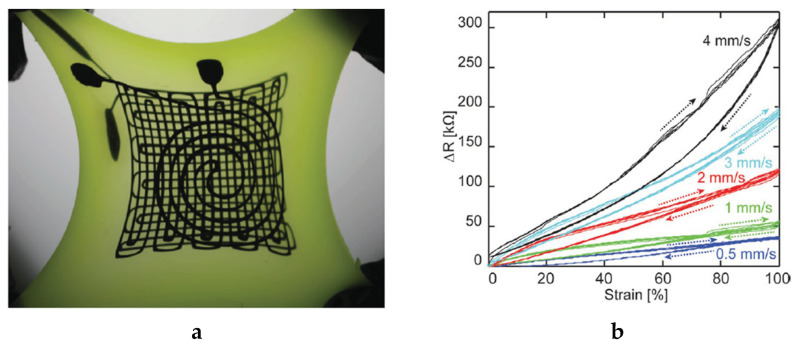
(**a**) Photograph of a three-layer strain and pressure sensor in the unstrained state (left) and stretched state (right). The top layer consists of a spiral pressure sensor, below which lies a two-layer biaxial strain sensor that consists of two square meander patterns (20 × 20 mm) oriented perpendicular to each other; (**b**) electrical resistance change as a function of elongation for sensors subjected to cyclic deformation, in which each sensor is cycled 5 times to 100% strain at a crosshead speed of 2.96 mm/s [144].

Recently, researchers are demanding that fabricating prototypes or producing complex structures can be done fast and at a low cost, but not many of the aforementioned fabrication techniques can offer this. However, as is well known, 3D printing can be a very cost-effective solution; as reported by [139], it can reduce lead times, improve the design, and/or lower the weight of the structure. Today, people are coming up with new and exciting uses for 3D printers all the time. Here are just a few examples that show what these machines can do. The airplane company Airbus is trying to figure out a way to make a 3D printer that is as big as an airplane hangar [140]. Currently, 3D printers use designs made on computers to make three-dimensional objects right before your eyes. For instance, Bellacicca et al. produced all-printed monolithic functional devices with designed 3D geometry and embedding passive electrical components [141]. Kuang et al. developed a 3D-printed shape memory elastomer that has potential application for biomedical devices, such as vascular repair devices, 3D printing of highly stretchable, shape-memory, and self-healing elastomer toward novel 4D printing [142]. Agarwalaa, et al. described the design, fabrication, and characterization of a microchannel-based strain sensor using flexible material [143]. The work explores the use of 3D printing, to fabricate the sensor in an easy and cost-effective way. It is shown that 3D printing can print complex designs with ease and fabricate objects with embedded features. Microchannels with dimensions of 500-micrometer diameter are printed within the sensor structure and filled with conductive silver nanoparticle ink. The printed sensor can measure normal (orthogonal to channels) and in-plane (parallel to channels) tensile forces and is tested using a custom-built test rig. Muth et al. also used 3D printing to develop a three-layer strain and pressure sensor within highly stretchable elastomers [144]. A multi-component materials system composed of ink, reservoir, and filler fluid were used to enable an e-3D printed strain and pressure sensor. A carbon conductive grease, i.e., carbon black particles in silicone oil functional ink was used to pattern the sensing elements. The actual image of the three-layer strain and pressure sensor is shown in Figure 22.

3D printing technology can be used to print objects through the use of a lot of materials. However, the objects have usually fixed geometrical structures, and are not helpful for multifunctional uses. For these reasons, researchers are also working to realize 4D printing in which stimuli-responsive active smart materials can be used to produce a 3D static structure [145]. The static structure is then able to convert or reconfigure into another new structure in the presence of a stimulus. Light, heat, pH, water, a magnetic field, or other means can be used as a stimulus based on the material chosen for the 3D printing. The concept behind 4D printing is what happens after the 3D printing is processed. A 4D material is able to transform from static, 3D structures into other smart objects that can grow, change shape, and move by themselves under a stimulus. Thus, 4D printing could be a promising approach to develop dynamic structures for smart textiles. To cope up with 4D printing, it is important to know about the chemistry and physics of smart materials and their behavior in-depth.

The concept of 4D printing may seem like something way beyond our time and factious, but many labs around the globe are already thriving to the futuristic prospects of this impressive approach. Most importantly, 4D printers do not exist as a separate or special functioning machine. Instead, the 3D printer is used to create the initial static object and include all the necessary 4D coding prior to subjecting the object to the elements that encourage the shape to vary. One of the main advantages of the concept of 4D is that you can create large 3D objects that would be too big to fit into an ordinary 3D printer. In the 4D printing technique, it should be possible to develop a smaller object in its first form which is then able to expand, bend, or fold-out into a larger object in its secondary form. 4D printing can be used on lots of different types of materials than originally thought, also textiles. Therefore, this approach could go to show that elements of science fiction are not too far away and lead to a new explosion of types of intelligent textiles.

In general, the development of 2D and 3D structures in conventional textiles will continue for integrated smart textiles, and 4D printing techniques will evolve further in the near future. Introducing new and advanced integration techniques will obviously speed up the realization of self-powered and computerized textiles; for example, to create smart clothing that makes soldiers invisible and invincible on the battlefield. Chemical, material, electrical, material, textile, computer, software, and medical experts should work in a team so that they can come up with efficient, effective, biocompatible, and long-lasting conductive materials and introduce scientific designs and integration approaches in/onto a textile structure, leading to a new generation of smart textile applications.

## 6. Conclusions

In this work, we have given a comprehensive review of the approaches of integrating electronic components on/into the textile structure. The review revealed that there are no specific processes that have been designed for smart textiles, instead, existing processes are being modified. It is convincible that the goal of smart textile development can only be achieved by using appropriate and convenient e-textile integration techniques. For that matter, all production technologies require further progress in all aspects. Knitting, weaving, embroidery, braiding, and laminating are mostly used, but the flexibility of the final product is unsatisfactory. Printing, plating, fiber spinning and coating methods are suitable if the starting conductive materials are a compound or ink. However, there is a technological challenge in printing thin conductive compounds on textile fabrics that have rough, uneven, or porous surfaces. Printing the entire components of the smart textile layer-by-layer via 3D printing and realizing 4D structures would lead to an evolution of completely new smart textile materials.

From a textile perspective, the overall aim for smart textiles is to convert all required components, like sensors, actuators, transmission lines, etc., into 100% textile material. To achieve this aim, we must tackle a big challenge from a technological point of view, that is, concepts, materials, and integration techniques must be made appropriate for use in, on, or as textile materials. Hence, the focus should be directed to improve the existing techniques and introducing new approaches that are able to cope with the advancement of material science and electronics.
